# Distinct characteristics of nasal polyps with and without eosinophilia^☆^

**DOI:** 10.1016/j.bjorl.2016.01.012

**Published:** 2016-04-22

**Authors:** Changzhi Sun, Hong Ouyang, Renzhong Luo

**Affiliations:** a Guangzhou Medical College, Guangzhou Women and Children's Medical Center, Department of Otolaryngology, Guangzhou, China; b China Three Gorges University, RenHe Hospital, Department of Otolaryngology, Yichang, China

**Keywords:** Nasal polyps, Th2 cells, Th17 cells, Pólipos nasais, Células Th2, Células Th17

## Abstract

**Introduction:**

Eosinophilic and noneosinophilic Nasal polyps (NPs) are different subtypes of NPs and require different treatment methods.

**Objective:**

To compare the histologic characteristics, mRNA and protein expression between Nasal Polyps with and without eosinophilia.

**Methods:**

NPs tissues were obtained from eighty-six NPs patients during surgery. Eosinophilic and noneosinophilic NPs were distinguished according to immunochemical results of the specimen. The histological, mRNA and protein expression features were compared between the two groups.

**Results:**

In eosinophilic NPs, we observed a significantly higher GATA-3, IL-5, IL-4, IL-13 mRNA and protein expression. In noneosinophilic NPs, IL-17, IL-23 and RORc mRNA and protein expression were increased. Immunohistochemistry tests showed, more mast cells and less neutrophils in eosinophilic NPs compared with noneosinophilic NPs. Eosinophilic NPs patient presented more severe symptom scores when compared to noneosinophilic NPs.

**Conclusion:**

We demonstrate for the first time that Th2 is the predominant reaction in eosinophilic NPs while Th17 is the predominant reaction in noneosinophilic NPs. Our study may provide new treatment strategy for NPs.

## Resumo

**Introdução:**

Pólipos nasais (PNs) eosinofílicos e não eosinofílicos são diferentes subtipos de PNs e requerem diferentes métodos de tratamento.

**Objetivo:**

Comparar as características histológicas e a expressão de mRNAs e proteínas entre PNs com e sem eosinofilia.

**Método:**

Amostras de PNs foram obtidos de 86 pacientes durante a cirurgia. PNs eosinofílicos e não eosinofílicos foram diferenciados segundo os resultados imunoistoquímicos de cada amostra. As características histológicas e de expressão de mRNAs e de proteínas foram comparadas entre os dois grupos.

**Resultados:**

Em PNs eosinofílicos, observamos uma expressão significativamente maior dos mRNAs e proteínas GATA-3, IL-5, IL-4 e IL-13. Nos PNs não eosinofílicos, aumentou a expressão dos mRNAs e proteínas IL-17, IL-23 e RORc. Nos testes imunoistoquímicos, observamos maior número de mastócitos e menor número de neutrófilos nos PNs eosinofílicos, em comparação com PNs não eosinofílicos. Os pacientes com PNs eosinofílicos obtiveram escores de sintomas mais graves *vs.* PNs não eosinofílicos.

**Conclusão:**

Demonstramos, pela primeira vez, uma reação Th2 predominante em PNs eosinofílicos e uma reação Th17 predominante em PNs não eosinofílicos. Nosso estudo pode proporcionar novas estratégias terapêuticas para a rinossinusite crônica.

## Introduction

Chronic rhinosinusitis (CRS) is characterized by persistent inflammation of nasal and paranasal mucosa, and is divided into two types according to the absence or presence of nasal polyps (NPs): CRS without NPs and CRS with NPs (CRSwNP).^1^ Histological features of NP include inflammation of Th2 cells accompanied by infiltration with eosinophils, thickening of basement membrane, and hyperplasia of the epithelium.^2^, ^3^, ^4^, ^5^ In Western populations, eosinophil infiltration is found in most NPs and considered as a major pathological marker of NPs.^6^, ^7^ However, more and more studies on Chinese NPs showed that many NPs patients in China presented as non-eosinophilic inflammation.^8^ For example, several studies have shown that a considerable proportion of Chinese NPs were neutrophil dominate, but the detailed difference between eosinophilic and non-eosinophilic NPs is still unknown.^9^

Similar studies on asthma have demonstrated that eosinophilic asthma is pharmacologically responsive to glucocorticoid, but non-eosinophilic asthma may be resistant to glucocorticoid.^10^, ^11^ In a recent study on the response of NPs patients to oral corticosteroid therapy conducted by Wen, they found eosinophilic NPs patients were more sensitive to corticosteroid compared with non-eosinophilic NP patients.^12^ Besides, eosinophilic NPs had a higher tendency of recurrence after surgery. All these studies demonstrated that eosinophilic and non-eosinophilic NPs may be different subtypes of NPs and needed different treatment methods.

This study aimed to investigate the expression of key transcription factors and cytokines for Th1/Th2/Th17 cells between eosinophilic and non-eosinophilic NPs and provide new information on CRSwNP.

## Methods

### Patients

Eighty-six patients with NPs were enrolled consecutively in this study. The diagnosis was mainly based on pathological examination. According to previous methods,^13^ eosinophilic and non-eosinophilic NPs were categorized based on immunochemical results by the presence of either <5 or ≥5 eosinophils/high powered fields (HPF), respectively. The baseline data were collected and Lund-Kennedy and Lund-Mackey score were obtained to evaluate the severity of NPs. None of the subjects used oral or nasal corticosteroids during four weeks before surgery. Details of all subjects are summarized in Table 1. This study was approved by the local ethics committee (No. 20130106) and informed consent was obtained.

**Table 1:** Demographic and clinical characteristics of eosinophilic and non-eosinophilic NP patients.

	Eosinophilic NP	Non-eosinophilic NP	*p*-value
Sex (male/female)	22/24	21/19	0.834
Age, years	41.3	39.4	0.235
Duration of symptoms, years	7.5	4.1	0.046
Absolute blood eosinophil count, 10^9^/L	0.28	0.11	0.013
Proportion of eosinophils (%)	2.6	7.8	0.003
Blood IgE level, kU/L	112	19	0.001
Patients with allergy (%)	65	22	0.002
Lund-Kennedy score	16.5	10.4	0.012
Lund-Mackey score	17.8	9.8	0.005
Time of surgery	2.3	1.1	0.042

NP, nasal polyp.

Every specimen was cut into two portions. One portion was stored at −80 °C for mRNA and protein analysis. The other portion was used for IHC staining.

### Symptom scores

At the clinical visit, the patients gave an overall assessment of their rhinitis symptoms. The symptoms of nasal blockage, nasal itching, sneezing, and rhinorrhea were rated on a 4-point scale, where 0 = no symptoms, 1 = mild, 2 = moderate, and 3 = severe. Total symptom scores ranged from 0 to 12 and represented the sum of the scores for nasal blockage, nasal itching, sneezing, and rhinorrhea.

### Immunohistochemical staining

For immunohistochemistry, the sections underwent dewaxing, dehydration, and then were placed in 0.3% H_2_O_2_ for 20 minutes at room temperature to reduce nonspecific background staining. After antigen retrieval by 10 mM citrate buffer for 15 minutes, antihuman monoclonal antibodies for MBP (eosinophils, 1:100, Santa Cruz), anti-HNE (neutrophils, 1:200, Dako) and anti-tryptase (mast cells, 1:100, Santa Cruz) were incubated overnight at 4 °C for immunohistochemical staining, respectively. The sections were washed with PBS and incubated with secondary antibody (Gene Tech – Shanghai, China) at room temperature for one hour on the next day.

After washing, DAB (Gene Tech, Shanghai, China) staining was performed under microscope and the sections were counterstained with Mayer's hematoxylin (Dako) for 40 s, dehydrated with series ethanol, cleared with xylene (three times), and mounted with neutral balsam (Dako). Control for nonspecific staining was routinely performed with PBS instead of primary antibodies, and all proved negative.

The sections were visualized with an Olympus CX40 Microscope (Olympus Europa, GmbH – Germany) and the number of positive cells was counted under high-power fields (400×). Ten fields were counted in each specimen and the median was calculated for each antibody.

### Real-time polymerase chain reaction (PCR) analysis

Real-time PCR were performed as previously described. Total mRNA was extracted from SIP or mucosa tissues using TRIzol reagent (Life Technologies–Carlsbad, CA, United States) according to the manufacturer's instructions. Reverse Transcription (RT) was performed, and cDNA was synthesized from 2 μg of total RNA using an oligo (dT) 18 primer and M-MLV reverse transcriptase (Takara–Syuzou, Shiga, Japan). The mRNA expression was determined using an ABI PRISM 7300 Detection System (Applied Biosystems–Foster City, CA, United States) and SYBR Premix Taq™ (Takara). The sequences of the primers are listed in Table 2. PRISM samples contained 1× SYBR Green Master Mix, 1.5 μL of 5 μM primers, and 25 ng of synthesized cDNA in a 25 μL volume. Reactions were heated to 95 °C for 10 min, followed by 40 cycles of denaturation at 95 °C for 10 s, and annealing extension at 60 °C for 60 s. All PCR reactions were performed in duplicate. Melting curve analysis was used to control for amplification specificity. The mean value of the replicates for each sample was calculated and expressed as a cycle threshold (Ct) value. The relative expression of each target gene was determined as the difference (ΔCt) between the Ct value of the target gene and the Ct value of β-actin. Fold changes in the target gene mRNA were determined as 2^−ΔΔCt^.

**Table 2:** Primers used for quantitative PCR analysis of transcriptional factors and cytokine gene expression.

Primer	Sequence
T-bet	(s) 59-GTCAATTCCTTGGGGGAGAT-39
	(a) 59-TCATGCTGACTGCTCGAAAC-39

GATA-3	(s) 59-CTGGCCACAGTTGTTTCATG-39
	(a) 59-GCAACTGGTGAACGGTAACA-39

RORC	(s) 59-GCTGTGATCTTGCCCAGAACC-39
	(a) 59-CTGCCCATCATTGCTGTTAATCC-39

IL-4	(s) 59-CCACGGACACAAGTGCGATA-39
	(a) 59-CCCTGCAGAAGGTTTCCTTCT-39

IL-5	(s) 59-CCCACAAGTGCATTGGTGAA-39
	(a) 59-CCTCAGAGTCTCATTGGCTATCAG-39

IL-17	(s) 59-CAAGACTGAACACCGACTAAG-39
	(a) 59-TCTCCAAAGGAAGCCTGA-39

IL-13	(s) 59-TGAGGAGCTGGTCAACATCA-39
	(a) 59-CAGGTTGATGCTCCATACCAT-39

IL-23	(s) 59-GAGCAGCAACCCTGAGTCCCTA-39
	(a) 59-CAAATTTCCCTTCCCATCTAATAA-39

GAPDH	(s) 59-ACCCAGAAGACTGTGGATGG-39
	(a) 59-TTCTAGACGGCAGGTCAGGT-39

PCR, polymerase chain reaction.

### Enzyme-linked immunosorbent assay (ELISA)

Freshly obtained tissue specimen was weighed, and protease inhibitor cocktail (Keygentec – Nanjing, China) was added per 100 mg of tissue. The tissue was then homogenized using homogenizer (Kinematica – Switzerland) for 1 min on ice. After homogenization, the suspension was centrifuged at 4000 rpm for 20 min at 4 °C, and the supernatants were stored at −80 °C until analyzed.

Enzyme-linked immunosorbent assay (ELISA) kits were used for measuring tissue levels of IL-5, IL-4, IL-13, IL-17, IL-23, IL-8, and MPO (R&D systems – Minneapolis, MN, United States) according to the manufacturer's protocols. Each sample was tested in duplicate. The detection limits of the assays were as follows: IL-5, 3.9 pg/mL, IL-4, 31.2 pg/mL, IL-13, 62.5 pg/mL, IL-17, 31.2 pg/mL, IL-23, 39 pg/mL, MPO, 1.56 mg/mL; and IL-8, 3.9 pg/mL.

### Statistical analysis

All data were expressed as mean ± SD. Statistical significance between two groups was determined using the Mann–Whitney *U* test. Significant difference was considered when *p* < 0.05.

## Results

### Subjects

Based on the histological criterion, 46 NP patients (53.5%) were classified into the eosinophilic subgroup and 40 NP patients (46.5%) were classified into non-eosinophilic subgroup. The demographic and clinical characteristics of patients are displayed in Table 1. Compared with non-eosinophilic NP patients, eosinophilic NP patients demonstrated a longer duration of symptoms, higher blood absolute eosinophil count, higher blood IgE level, higher percentage of allergic history, higher proportion of eosinophils, higher Lund-Kennedy and Lund-Mackey score, more incidence of surgery, and higher symptom scores. In term of age and sex, there was no significant difference between eosinophilic and non-eosinophilic NP patients.

### Comparison of histology between eosinophilic and non-eosinophilic NPs

It was found that the number of total inflammatory cells in eosinophilic NPs increased significantly compared with non-eosinophilic NPs (data not shown). Regarding cell types, eosinophilic NPs have increased mast cells except for eosinophils, whereas the non-eosinophilic NP presented more neutrophil infiltration (Table 3). The results also showed that 85% of eosinophilic NPs had more than six mast cells per HPF, while all non-eosinophilic NPs had less than three mast cells per HPF. Meanwhile, non-eosinophilic NPs presented as severe neutrophil infiltration. 90% of non-eosinophilic NPs had more than five mast cells per HPF, while all eosinophilic NPs had less than two neutrophil per HPF. When the relationship between the number of eosinophils and other cells was also analyzed, it was found that the number of eosinophils correlated positively with the number of mast cells (*r* = 0.68; *p* < 0.001) and total inflammatory cells (*r* = 0.46; *p* < 0.05), but no relationship was found between eosinophils and neutrophils.

**Table 3:** Median score and 95% reference range (mean ± SEM) of cell counts in eosinophilic and non-eosinophilic NP patients (per high-powered field).

	Eosinophilic NP	Non-eosinophilic NP	*p*-Value
Eosinophil	9.3 (8.8 ± 2.1)	2.8 (2.6 ± 0.3)	0.02
Neutrophil	1.5 (1.7 ± 0.5)	6.3 (5.4 ± 1.5)	0.04
Mast cell	7.6 (6.8 ± 2.3)	2.9 (2.8 ± 0.1)	0.01

NP, nasal polyps.

### Comparison of mRNA expression between eosinophilic and non-eosinophilic NPs

**Figure 1:**
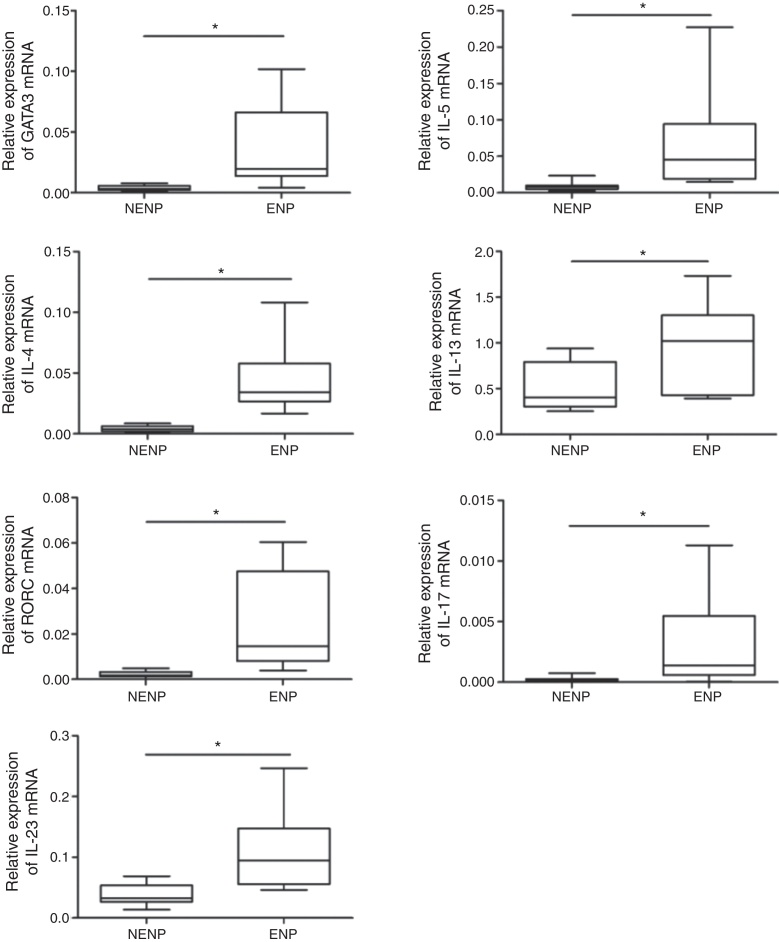
Comparison of mRNA expression between eosinophilic and non-eosinophilic nasal polyps. NENP, non-eosinophilic nasal polyps; ENP, eosinophilic nasal polyps.

In eosinophilic NPs, significantly higher Th2 (GATA-3, IL-5, IL-4, IL-13) transcription factor and cytokines expression were observed, while in non-eosinophilic NPs, Th17 (RORC, IL-17A, and IL-23) transcription factor and cytokines showed increased expression (Fig. 1). For Th1 (T-bet and IFN-γ) transcription factor and cytokines, no difference was found between two groups (data not shown).

### Comparison of protein expression between eosinophilic and non-eosinophilic NPs

**Figure 2:**
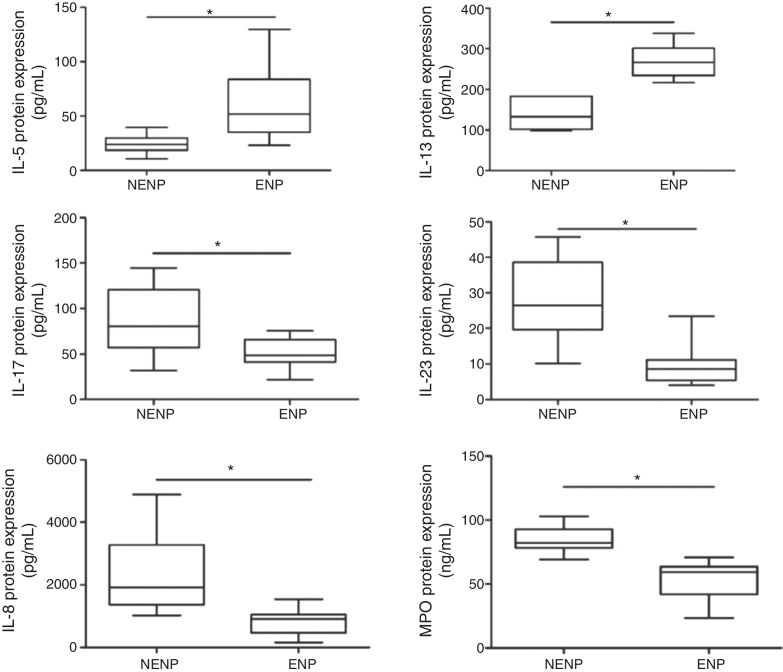
Comparison of protein expression between eosinophilic and non-eosinophilic NPs. NENP, non-eosinophilic nasal polyps; ENP, eosinophilic nasal polyps.

Similar with the mRNA expression, Th2 cytokines (IL-5 and IL-13) protein were significantly higher in eosinophilic NPs and Th17 cytokines (IL-17A and IL-23) protein were significantly higher in non-eosinophilic NPs (Fig. 2). However, IL-4 protein expression was undetectable in both groups (data not shown). As for Th1 cytokines (IL-12 and IFN-γ) expression, no difference was found between two groups (data not shown). It was also found that expression of markers of neutrophil (IL-8, MPO) in non-eosinophilic NPs was higher than that of eosinophilic NPs. The relationship between cytokine expression was also analyzed; it was found that IL-17A was positively related to both IL-8 and MPO expression (*r* = 0.53, *p* < 0.01; *r* = 0.57, *p* < 0.05).

## Discussion

In Western populations, NPs are considered to be orchestrated by Th2 cells, and tissue eosinophilia is a very important feature.^14^, ^15^, ^16^ The infiltration and activation of eosinophils in nasal mucosa can promote secretion of specific granule proteins, synthesis, and release of lipid mediators, inflammatory cytokines, chemokines, and growth factors. Through these chemical mediators, eosinophils contribute to the development of NPs. However, several studies have demonstrated that less than 50% of NP patients in China or other Asian countries had eosinophilic inflammation.^17^, ^18^ These findings indicate that NPs are heterogeneous and can be broadly divided into two subtypes: eosinophilic and non-eosinophilic NPs. Eosinophilic NPs can be well-controlled by corticosteroid therapy, while non-eosinophilic NPs are responsive to a combination of endoscopic sinus surgery and macrolide therapy.^19^, ^20^, ^21^

In the present study, the eosinophilic NP group demonstrated a higher prevalence of allergy and IgE levels. Since blood eosinophil count is significantly correlated with eosinophil infiltration in the nasal polyps, the blood eosinophil count could be a good marker for the eosinophilic inflammation of NPs. All these data suggested that the occurrence of eosinophilic NPs was closed related to allergy. However, the role of allergy in the pathogenesis of NPs is still controversial. A few studies have questioned the role of allergy in the pathogenesis of NPs. Caplin et al. evaluated 3000 atopic patients and found that only 0.5% of patients had NPs. Other reports were also unable to support either a higher incidence of atopy in patients with NPs or a pattern of allergic inflammation in the pathogenesis of NPs.^22^, ^23^, ^24^ Thus, the present study requires further validation by studies with larger sample size. It was also found that eosinophil infiltration was directly correlated with disease severity, since both total and each item's score were higher in eosinophilic NPs. Besides, higher Lund-Kennedy and Lund-Mackey scores in eosinophilic NPs were also found. Taken together, these results suggest that eosinophilic NPs predict long disease duration and poor prognosis.

In the histological test, it was found that more severe inflammatory reaction in eosinophilic NPs presented as more mast cells and eosinophils infiltration, and more neutrophil infiltration in non-eosinophilic NPs. Mast cells are the major effector cell of IgE-mediated allergic reactions by releasing histamine and other chemicals involved in allergic inflammation; their infiltration into eosinophilic NPs suggested that a T-cell-mediated immune response may play an important role in eosinophilic NPs. Consistent with these results, the data showed that the number of eosinophils was positively correlated with the number of mast cells and total inflammatory cells. Neutrophil infiltration is often believed to be related with bacterial infection, but the present results demonstrated that neutrophil infiltration was also involved in chronic inflammation. Nagakura suggested that high-molecular-weight neutrophil chemotactic activity was related to nasal hypersensitivity. In addition, elastase released by neutrophils after degranulation may also play an important role in tissue damage.^25^, ^26^

Previous studies had confirmed the role of Th cell in the pathogenesis of CRS, thus, the present study compared the expression of mRNA and protein expression between two groups. The results revealed Th2 predominant reaction in eosinophilic NP and Th17 predominant reaction in non-eosinophilic NP. However, Th1 (T-bet and IFN-γ) transcription factors and cytokines expression were found between the two groups. GATA-3 is necessary for commitment toward Th2 cells and controls the expression of IL-5.^27^, ^28^, ^29^, ^30^ The upregulation of GATA-3 in eosinophilic NP was reflected by the subsequent increase of the IL-5 mRNA and protein. As a marker for Th17 cells, the transcription factor RORC was analyzed and significantly higher expression was found in non-eosinophilic NPs.^31^, ^32^ Similarly, Th17 cytokines (IL-17A and IL-23) protein were significantly higher in non-eosinophilic NPs. Consistent with these results, previous studied have shown that the IL-17-IL-23 axis plays important roles in the pathogenesis of NPs. As expected, markers of neutrophils (IL-8, MPO) in non-eosinophilic NPs were higher than that of eosinophilic NPs. It was also found that IL-17A was positively related to both IL-8 and MPO expression, suggesting that IL-17 may be related to neutrophil migration; its mechanism requires further exploration.

## Conclusion

In summary, the data demonstrate, for the first time, Th2 predominant reaction in eosinophilic NPs and Th17 predominant reaction in non-eosinophilic NPs. This study may provide new treatment strategy for NPs.

## Ethical standards

The authors assert that all procedures contributing to this work comply with the ethical standards of the relevant national and institutional guidelines on human experimentation and with the Helsinki Declaration of 1975, as revised in 2008.

## Funding

Supported by Medical Scientific Research Foundation of Guangdong Province, China, A2013518.

## Conflicts of interest

The authors declare no conflicts of interest.
